# Beyond greenness: Detecting temporal changes in photosynthetic capacity with hyperspectral reflectance data

**DOI:** 10.1371/journal.pone.0189539

**Published:** 2017-12-27

**Authors:** Mallory L. Barnes, David D. Breshears, Darin J. Law, Willem J. D. van Leeuwen, Russell K. Monson, Alec C. Fojtik, Greg A. Barron-Gafford, David J. P. Moore

**Affiliations:** 1 School of Natural Resources and the Environment, University of Arizona, Tucson, Arizona, United States of America; 2 Department of Ecology and Evolutionary Biology, University of Arizona, Tucson, Arizona, United States of America; 3 School of Geography and Development, University of Arizona, Tucson, Arizona, United States of America; 4 Department of Geology, Wheaton College, Wheaton, Illinois, United States of America; University of Maryland at College Park, UNITED STATES

## Abstract

Earth's future carbon balance and regional carbon exchange dynamics are inextricably linked to plant photosynthesis. Spectral vegetation indices are widely used as proxies for vegetation greenness and to estimate state variables such as vegetation cover and leaf area index. However, the capacity of green leaves to take up carbon can change throughout the season. We quantify photosynthetic capacity as the maximum rate of RuBP carboxylation (V_cmax_) and regeneration (J_max_). V_cmax_ and J_max_ vary within-season due to interactions between ontogenetic processes and meteorological variables. Remote sensing-based estimation of V_cmax_ and J_max_ using leaf reflectance spectra is promising, but temporal variation in relationships between these key determinants of photosynthetic capacity, leaf reflectance spectra, and the models that link these variables has not been evaluated. To address this issue, we studied hybrid poplar (*Populus* spp.) during a 7-week mid-summer period to quantify seasonally-dynamic relationships between V_cmax_, J_max_, and leaf spectra. We compared *in situ* estimates of V_cmax_ and J_max_ from gas exchange measurements to estimates of V_cmax_ and J_max_ derived from partial least squares regression (PLSR) and fresh-leaf reflectance spectroscopy. PLSR models were robust despite dynamic temporal variation in V_cmax_ and J_max_ throughout the study period. Within-population variation in plant stress modestly reduced PLSR model predictive capacity. Hyperspectral vegetation indices were well-correlated to V_cmax_ and J_max_, including the widely-used Normalized Difference Vegetation Index. Our results show that hyperspectral estimation of plant physiological traits using PLSR may be robust to temporal variation. Additionally, hyperspectral vegetation indices may be sufficient to detect temporal changes in photosynthetic capacity in contexts similar to those studied here. Overall, our results highlight the potential for hyperspectral remote sensing to estimate determinants of photosynthetic capacity during periods with dynamic temporal variations related to seasonality and plant stress, thereby improving estimates of plant productivity and characterization of the associated carbon budget.

## Introduction

Photosynthesis by land plants plays a critical role in regional and global carbon balance [1–3]. Globally, photosynthesis in the terrestrial biosphere, combined with photosynthetic and non-photosynthetic processes in the oceans, offsets 45% of anthropogenic carbon emissions annually [4]. Terrestrial carbon uptake by plants varies annually and seasonally based on climate conditions [5–9], water and nutrient availability [10,11], and plant physiological properties such as water use efficiency [12] and photosynthetic capacity [13]. The size and future of the terrestrial carbon sink remains a critical uncertainty in global climate models [14]. To accurately predict terrestrial carbon uptake, improved quantification of spatial and temporal variation in photosynthesis is necessary.

Spatial and temporal variation in plant photosynthesis can be estimated using remote sensing-derived spectral indices. Spectral estimates of green vegetation, including vegetation indices such as the Normalized Difference Vegetation Index (NDVI), are widely used to estimate photosynthesis and vegetation productivity across spatial and temporal scales [15–18]. Estimates of gross primary productivity derived from greenness measures rely on relationships between vegetation use of light energy and photosynthesis [19–22]. However, the capacity of green leaves to use absorbed light to convert CO_2_ into biomass varies dynamically throughout the season and with plant stress [23–26]. Many terrestrial biosphere models use static values of determinants of photosynthetic capacity [27]; however, there is some evidence that allowing photosynthetic capacity to vary temporally could improve representation of carbon dynamics [28]. The photochemical reflectance index (PRI) is a hyperspectral vegetation index that detects diurnal changes in xanthophyll cycle activity and responds to seasonal shifts in leaf pigment concentrations [29]. PRI is used to estimate photosynthetic light-use efficiency [30]; however, its relationship to photosynthetic capacity is unclear. Spectral methods that capture dynamic temporal changes in photosynthetic capacity could yield more accurate estimates of vegetation productivity and associated carbon uptake.

Photosynthetic capacity represents the potential of vegetation to fix CO_2_ under optimal light and water conditions. In this study, we estimate photosynthetic capacity through measurements of the maximum rate of carboxylation of RuBP by the enzyme rubisco (V_cmax_) and the maximum rate of electron transport driving RuBP regeneration (J_max_)[31]. Some terrestrial biosphere models treat V_cmax_ and J_max_ as fixed parameters [27]. However, these photosynthetic parameters vary in response to climate conditions [32–35], atmospheric CO_2_ concentrations [36], plant stress [37], and seasonally in response to ontogenetic processes [38–40]. This seasonal variation can be important, given that process-based biosphere models that account for within-season variation in photosynthetic capacity show improved predictions of carbon flux dynamics [28].

Although hyperspectral remote sensing shows promise for predicting photosynthetic capacity based on leaf optical properties, questions remain regarding temporal variability. Estimates of photosynthetic capacity from remote sensing methods are desirable because of their potential to map V_cmax_ and J_max_ across space and constrain terrestrial biosphere model estimates of plant function. Predictive models of photosynthetic capacity, conditioned based on leaf reflectance metrics, have been developed using partial least squares regression (PLSR) for multiple tree species across glasshouse temperature regimes [29], in C4 crop species[41], and in C3 species at the canopy level [33]. Emergent studies on crop species using high-throughput phenotyping approaches further justify a better understanding of hyperspectral characterization of photosynthetic capacity [41–43]. Still unknown is whether hyperspectral methods of estimating V_cmax_ and J_max_ are robust to temporal variation in these key determinants of photosynthetic capacity. Hyperspectral leaf reflectance correlates with leaf characteristics likely to cause seasonal variation in photosynthetic capacity such as chlorophyll content [44], nitrogen content [45], light-use efficiency [46], and water status [47], while spectral vegetation indices capture some but not all of these factors. However, previous studies have generally used variation in reflectance and plant function across space rather than time to derive these relationships so it remains unknown whether hyperspectral methods of estimating V_cmax_ and J_max_ are robust to temporal variation in these key determinants of photosynthetic capacity. We hypothesize that if seasonal variation in photosynthetic capacity is caused by a combination of leaf changes detectable by reflectance in bands outside of narrowband multispectral bands, then PLSR models that utilize the full reflectance spectrum will predict seasonal changes in V_cmax_ and J_max_.

In this study, we evaluated the ability of hyperspectral data to represent and predict within-season temporal variation in V_cmax_ and J_max_ and examined the influence of water stress on the robustness of these estimates of photosynthetic capacity. We compared leaf reflectance spectra from hybrid poplar (*Populus* spp.*)* to V_cmax_ and J_max_ estimates throughout a 7-week period in the middle part of the growing season. We discuss our results in the context of emerging hyperspectral remote sensing methods and terrestrial biosphere models of global carbon dynamics.

## Methods

### Experimental site

Our study was conducted using poplar trees, grown outdoors at the Biosphere 2 Research Center near Oracle, AZ, USA (32° 34’ 51” N 110° 50’ 57” W; 1189 m). Biosphere 2 is leased to the University of Arizona. Authors RKM, DJPM, and GABG were responsible for the poplar stand at Biosphere 2. No additional permission was required to carry out this study, which did not involve endangered or protected species. We studied the relationship between spectral reflectance and photosynthetic capacity in 12 individual *Populus deltoides* hybrid poplar trees. Trees were planted in random arrangement with 1 x 1 m spacing in January 2013. Each year during the dormant season, the trees were coppiced and destructively sampled for biomass. Study trees were randomly selected before the start of the experiment in May 2016. During the study period (5/24/2016–7/5/2016), the mean high temperature was 34.4°C and the mean low temperature was 21.3°C. We applied 38 liters of water per day per tree during the pre-dawn period for 2 weeks prior to the start of the experiment (5/1/2016–5/14/2016) using an irrigation system to begin the study in well-watered conditions. We also fertilized the trees on 5/14/2016 using tree and shrub food (Arizona’s Best) to ensure the trees were not nutrient-limited at the start of the induced dry-down. The trees were exposed to ambient climate conditions and had no irrigation except for supplemental watering from 6/2/2016 through 6/6/2016 and additional watering on 6/20/2016, which avoided senescence and caused variance in the water status of the trees. On sampling dates, we measured predawn leaf water potential (Ψ_pd_), leaf gas exchange, and hyperspectral leaf reflectance for each tree. The full suite of measurements was conducted on all 12 trees on consecutive sampling dates (6 trees per day), except for 6/30 when we measured all 12 trees in one day.

### Predawn leaf water potential and A/Ci curves

Predawn leaf water potential (Ψ_pd_) was measured using a pressure chamber (PMS Instruments, Albany, OR, USA). Leaves were collected before sunrise, transported to the lab in a cooler, and measured within 30 minutes of collection. One leaf per plant was measured per time point.

Leaf gas exchange was measured with two LI-COR portable photosynthesis systems (LI-COR Biosciences, Lincoln, NE, USA) equipped with a 6400-02B LED light source. Gas exchange measurements were performed on the youngest, most fully-expanded leaf on the south-facing side of each tree. Leaves were acclimated to the chamber at 25°C, a chamber-air CO_2_ concentration of 400 ppm, and a saturated photosynthetic photon flux density (PPFD) of 1800μmol m^-2^ s^-1^ until the photosynthetic rate (A) stabilized. Gas exchange curves were conducted only on leaves with an initial A ≥ 10 μmol CO_2_ m^-2^ s^-1^ to ensure that the leaves were active enough to yield appropriate estimates for photosynthetic capacity. Each curve consisted of 13 different intercellular CO_2_ concentrations (Ci) starting at the ambient CO_2_ concentration of 400 ppm and then decreasing to 300, 200, 100, 50 to 0 ppm before increasing to 400, 400, 600, 800, 1200, 1600, 2000 ppm. We used the Predictive Ecosystem Analyzer (PEcAn) photosynthesis package to perform quality control of CO_2_ response data before fitting A/Ci curves (https://github.com/PecanProject/pecan). In total, 86 CO_2_ response curves passed quality control. We fit A/Ci curves using the ‘fitaci’ function in ‘plantecophys’ package in R [48]. The ‘fitaci’ function fits the Farquhar-Berry-Von Caemmerer Model of leaf photosynthesis [31] to measurements of photosynthesis and intercellular CO_2_ and estimates V_cmax_ and J_max_ along with their standard errors.

### Hyperspectral measurements

Reflectance was measured on the same leaves as the gas exchange measurements using a high-spectral resolution ASD FieldSpec® 3 Full-Range (350–2500 nm) spectroradiometer (Analytical Spectral Devices, Boulder, CO, USA). Reflectance measurements were taken with a leaf-clip assembly containing an internal calibrated light source and a black background panel face. The relative leaf reflectance data were standardized prior to measurements of each leaf by measuring a standard white reference reflectance target. ViewSpec Pro® software was used to convert binary data to ASCII data. The spectral resolution is 3nm at 700 nm, 10nm at 1400 and 2100nm across the full spectrum. The hyperspectral data are sampled at every 1nm. The spectroradiometer was turned on for at least 30 minutes before reflectance measurements were taken. The white panel reference reflectance was captured every 2–5 minutes. All measurements were taken from the leaf adaxial surface, avoiding the midrib. Three reflectance measurements were obtained on three different areas of each leaf lamina, resulting in nine spectra per leaf. Each measurement required no more than 5 s. Spectral reflectance measurements were quality-checked by removing negative reflectance values. We then averaged the nine spectra to determine mean optical properties for each leaf. Measures of leaf optical properties generally occurred between 10:30am and 11:30am and followed gas exchange measurements by no more than 2 hours.

### Partial least squares regression models

Partial least-squares regression (PLSR) was performed to generate predictive models using the package ‘PLS’ in R [49]. PLSR is a multivariate regression method commonly used in spectroscopy because it can account for many related predictor variables and relatively few observations. PLSR identifies key components that explain variation in a trait variable and generates a linear model to transform full-spectrum data based on these components. The package ‘PLSROpt’ in R was used for pre-processing the spectral data in the order of standard normal variate, a second-derivative Savitzky-Golay smoother, auto-scaling, and mean centering (https://github.com/uwadaira/plsropt). The model with the number of components that minimized the Root Mean Squared Error of Prediction (RMSEP) was selected as the most parsimonious PLSR model. Each PLSR model was generated independently for V_cmax_ and for J_max_. The spectrum range for all models was 450–2500 nm. Performance parameters were generated to assess the predictive ability of each model including the coefficient of determination (R^2^).

Three different evaluations of the PLSR model were performed in increasing order of statistical rigor. The first test was a "leave-one-out" cross-validation approach, which trains the model on all but one observation, and then makes a prediction based on the single remaining observation [50]. The second test was performed with a 20% holdout dataset; a random 80/20% split of the data divided it into a training and testing dataset, respectively. New PLSR models were generated based on calibration of the training dataset and validated based on the remaining 20%. A 100x cross validation of training/calibration splits was performed to assess model and data stability across different proportions of testing and training data (S1 Fig). The third and most rigorous test of model stability was testing the model on the sampling dates with greatest variation in the population in terms of water stress (Ψ_pd_). The two consecutive sampling dates with the largest individual variation in Ψ_pd,_ (6/23/2016 and 6/24/2016, n = 12) were held out. The model was trained on all data except for the holdout dataset and was tested on the 12 observations with large variation in Ψ_pd._ The purpose of this test was to assess model predictive capacity in a situation with large within-population variation in environmental stress. For each PLSR model, Selectivity Ratio (SR) scores were calculated to enable comparison of the relative significance of each wavelength in its contribution to the final model. Although the Variable Importance of the Projection (VIP) score is more widely used in the current scientific literature, SR has been found to be more reliable for model predictions and is thus presented in this study [51].

### Spectral vegetation indices

Spectral vegetation indices for estimating chlorophyll content, water stress, and carotenoid pigments were tested using the full set of observations (Table 1); these are all published indices used to estimate plant physiological status. The Normalized Difference Water Index (NDWI) was tested based on its correlation with plant water content in conifers [47], and PRI was tested based on known relationships with photosynthetic functioning [52,53] and environmental stress [54,55]. We also calculated ‘MODIS-like’ NDVI for all spectra. MODIS (Moderate Resolution Imaging Spectroradiometer) NDVI uses the red band (band 1; 620–670 nm) and the near-infrared (NIR) band (band 2; 841–876 nm). For both the red and NIR, we calculated the full-width- half-maximum for the subset of wavelengths corresponding to the MODIS bandwidths using the ‘peakshape’ function in the ‘pavo’ package [56]. We then took the mean of all reflectance values between the endpoints of the width of the spectrum curve. We then applied the standard NDVI equation (see Table 1) to get a ‘MODIS-like’ NDVI. Pairwise correlations between V_cmax_ and J_max_ estimated using standard gas exchange techniques and spectral indices were tested and R^2^ reported.

**Table 1:** Hyperspectral vegetation indices that were compared to estimated V_cmax_ and J_max_ values.

Index	Formula	Reference	R^2^ Vcmax	R^2^ Jmax
SR1	ρ750/ρ700	Gitelson and Merzlyak 1997 [57]	**0.74**	**0.61**
Double Difference	(ρ749-ρ720)-(ρ701-ρ672)	le Maire et al. 2004 [58]	**0.74**	**0.58**
Vogelmann1	ρ740/ρ720	Vogelmann et al. 1993 [59]	**0.73**	**0.58**
mSR705	(ρ750-ρ445)/(ρ705-ρ445)	Sims and Gamon 2002 [44]	0**.73**	**0.57**
SRCarter	ρ760/ρ695	Carter et al. 1994 [60]	**0.72**	**0.61**
Maccioni	(ρ780-ρ710)/(ρ780-ρ680)	Maccioni et al. 2001 [61]	**0.72**	**0.55**
SR3	ρ750/ρ550	Gitelson and Merzlyak 1997 [57]	**0.70**	**0.63**
Gitelson	1/ρ700	Gitelson et al. 1999 [62]	**0.62**	**0.52**
NDVI (MODIS-like)	(ρ NIR_MODIS_ - ρ Red_MODIS_)/(ρ NIR_MODIS_ + ρ Red_MODIS_)	*see methods*	**0.60**	**0.50**
Datt4	ρ672/(ρ550*ρ708)	Datt (1998) [63]	**0.57**	0.45
SR4	ρ700/ρ670	McMurtey et al. (1994)	**0.57**	0.36
SR2	ρ752/ρ690	Gitelson and Merzlyak 1997 [42]	**0.56**	**0.53**
NDVI (hyperspectral)	(ρ860-ρ690)/(ρ860+ρ690)	Stimson et al. 2005 [47]	0.49	0.46
Vogelmann2	(ρ734-ρ747)-(ρ715+ρ726)	Vogelmann et al. 1993 [59]	0.17	0.14
mNDVI	(ρ800-ρ680)/(ρ800+ρ680–2ρ445)	Sims and Gamon 2002 [44]	0.07	0.03^ns^
NDWI	(ρ860-ρ1240)/(ρ860+ρ1240)	Gao 1996 [64]	0.06	0.20
SIPI	(ρ800-ρ445)	Penuelas et al. 1995 [65]	0.06	0.03^ns^
PRI	(ρ531-ρ570)/(ρ531+ρ570)	Gamon 1997 [52]	0.02^ns^	0.00^ns^
mSRCHL	(ρ800-ρ445)/(ρ680-ρ445)	Sims and Gamon 2002 [44]	0.00^ns^	0.00^ns^

The predictive formula (ρ = spectral reflectance) and reference for each index is shown in addition to the coefficient of determination for V_cmax_ and J_max_. Nonsignificant correlations are indicated with an “ns” superscript. Values of R^2^ above 0.50 are bolded.

## Results

**Fig 1:**
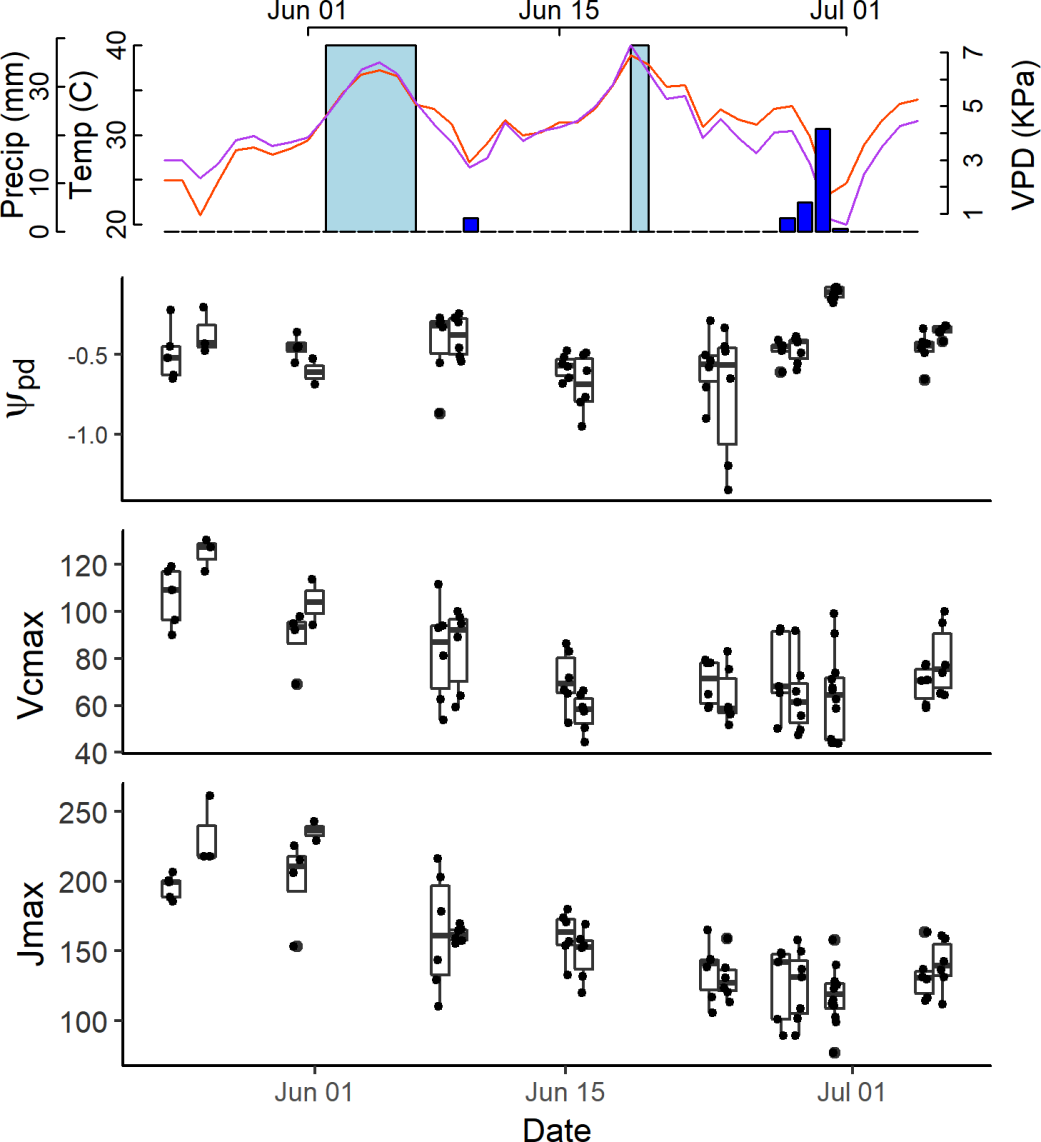
Temporal variability in meteorological and physiological conditions throughout the study period. A) shows mean daytime temperature in °C (orange line; 06:00 to 18:00), peak VPD in kPa (purple line; 10:00 to 14:00), precipitation in mm and supplemental watering throughout the study period with dark blue bars representing precipitation and light blue representing supplemental watering days. B) Shows the time series of predawn water potential (MPa) throughout the study period. C) and D) show the time series of estimated V_cmax_ (μmol m^-2^ s^-1^) and J_max_ (μmol m^-2^ s^-1^), respectively.

**Fig 2:**
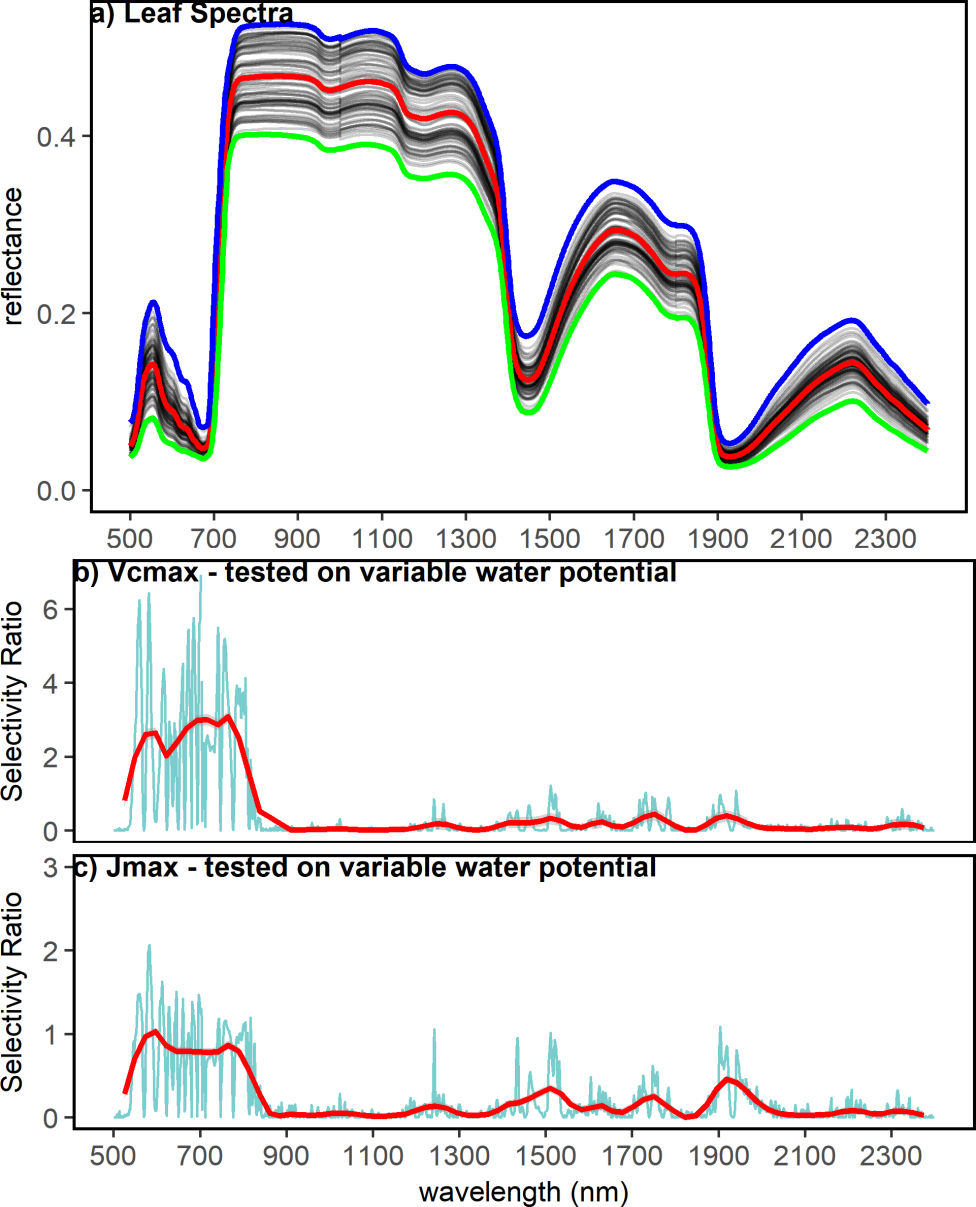
Leaf reflectance spectra and wavelength importance in PLSR models. (A) Pooled reflectance spectra (λ = 450–2500 nm) for all plants studied throughout the course of the study period (5/24/2016 to 7/05/2017). The mean reflectance at each wavelength is shown by the red line, the maximum and minimum reflectance at each wavelength are shown by the blue and green lines, respectively. (B)&(C): selectivity ratio of each wavelength in the PLSR models tested on variable water potential. (B) Shows the selectivity ratio of each wavelength in the V_cmax_ PLSR model, and (C) shows the selectivity ratio of each wavelength in the J_max_ PLSR model. The red lines in B&C are LOESS smoothers for visualization purposes.

Weather conditions, photosynthetic capacity, and pre-dawn water potential (Ψ_pd_) varied over the course of our study (Fig 1). Conditions were generally hot and dry throughout the study period, with high daytime temperatures and Vapor Pressure Deficit (VPD) and low precipitation (Fig 1A). Mean daytime temperature (06:00 to 18:00 MST) ranged from 20.73°C to 38.90°C throughout the study period with a mean of 30.1 ±4.3°C. Peak VPD (10:00 to 14:00 MST) ranged from 0.60 kPa to 7.2 kPa with a mean of 3.9 ±1.3 kPa. There was little precipitation throughout the study period with most (30.3 mm) of the total rainfall (36.1 mm) occurring between 6-28-2016 and 6-30-2016 (Fig 1A). V_cmax_ and J_max_ both varied throughout the study period. V_cmax_ ranged from 43.9 to 130.4 with a mean of 75.7 ±20.8 μmol m^−2^ s^−1^. J_max_ ranged from 76.7 to 261.2 with a mean of 150.1 ±37.5 μmol m^−2^ s^−1^. V_cmax_ and J_max_ declined by approximately two-fold throughout the 7-week study period (Fig 1C and 1D). V_cmax_ and J_max_ appeared to stabilize following the rain events between 6-28-2016 and 6-30-2016 which relieved water stress (Fig 1B). Pre-dawn water potential varied seasonally, with the lowest water potentials (most stressed) generally occurring in the middle of the study period (6–23 and 6–24, Fig 1B). Water potential values ranged from -1.35 to -0.8 MPa with a mean of -0.45 ±0.23 MPa. Leaf reflectance varied between individuals and temporally (Fig 2A). We evaluated our results first in terms of variability in leaf reflectance spectra in the study period, then evaluated estimates of V_cmax_ and J_max_ from three different variations of PLSR models with increasing statistical rigor, and finally assessed relationships between V_cmax_ and J_max_ and published hyperspectral indices.

**Fig 3:**
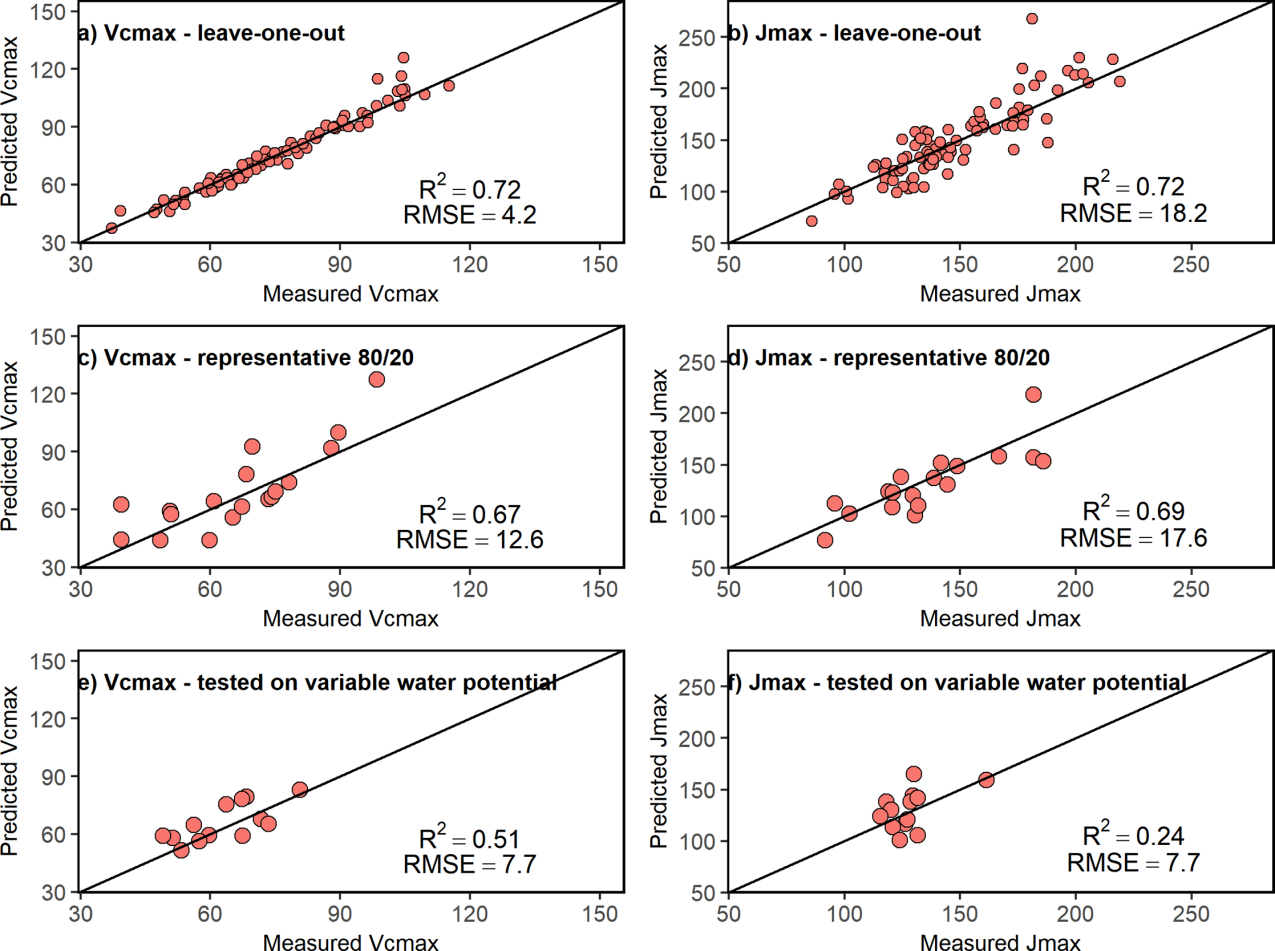
Temporal stability of PLSR models. All figures show observed vs. predicted values from PLS regression models. A&B: leave-one-out cross-validation procedure for (a) maximum rates of RuBP carboxylation (V_cmax_, μmol m^-2^ s-^1^) and (b) RuBP regeneration J_max_ (μmol m^-2^ s-^1^), n = 86. C&D: representative 80/20% split of the data for PLSR (C) V_cmax_ (μmol m^-2^ s-^1^) and (D) J_max_ (μmol m^-2^ s-^1^). Each model was trained on 69 observations and tested on 17 observations. E & F: models tested on the period with the greatest between-individual variation in pre-dawn water potential (Ψ_pd_) for (E) V_cmax_ (μmol m^-2^ s-^1^) and (F) J_max_ (μmol m^-2^ s-^1^). Each model was trained on 74 observations and tested on the 12 observations on 6/23/2016 and 6/24/2016 when the population varied widely in Ψ_pd_. Statistical tests of increase in rigor moving downward.

We performed three different evaluations of the PLSR models, presented in increasing order of statistical rigor. First, to assess the relationship between photosynthetic capacity and leaf spectra, PLSR models based on the complete dataset were developed. This least rigorous test of temporal stability used the "leave-one-out" cross validation approach to quantify the relationship between leaf reflectance spectra (450–2500 nm) and V_cmax_ and J_max_. PLSR models predicted photosynthetic capacity accurately, with comparable model predictive ability for V_cmax_ (R^2^ = 0.72; Fig 3A) and J_max_ (R^2^ = 0.72; Fig 3B). The root mean squared error (RMSE) was lower for the V_cmax_ model (RMSE = 4.2, Fig 3A) than the J_max_ model (RMSE = 18.2, Fig 3B). The predictive model for V_cmax_ only required two components to explain the variance, while the PLSR model for J_max_ required four components.

Second, to assess temporal stability of the model, the data were split into training and testing datasets to generate PLSR models. This second test of PLSR model stability used 80% of the observations for training and withheld a 20% holdout dataset for testing. The 20% holdout dataset was randomly selected from the full dataset and the remaining 80% were used to build PLSR models for V_cmax_ and J_max_. This procedure was performed 100 times with different randomly selected 20% holdout datasets (S1 Fig); the mean R^2^ for the 80/20 split was 0.64 ±0.03 for V_cmax_ and 0.64 ±0.07 for J_max_ (S1 Table). Models with a 20% holdout dataset that were representative of mean predictive ability are shown in Fig 3C and 3D; predictive ability was comparable for V_cmax_ (R^2^ = 0.67; Fig 3C) and J_max_ (R^2^ = 0.69; Fig 3D). The predictive capability of the 80% model was similar to the predictive capability of the full cross-validated model (Fig 3A and 3B). The RMSE was lower for the V_cmax_ model (RMSE = 12.6; Fig 3C) than the J_max_ model (RMSE = 17.6; Fig 3D).

Third, to assess temporal stability in situations with large within-population variation in environmental stress, the data was split into a testing and training set based on variability in Ψ_pd_. This third and most rigorous test of PLSR model stability trained the PLSR model on low environmental stress conditions (Ψ_pd_) and tested it on the two consecutive sampling days with highest individual variation in Ψ_pd,_ (6/23 and 6/24; Fig 1C). PLSR models for V_cmax_ and J_max_ in this third test differed in their predictive abilities. The predictive ability was only moderately reduced for V_cmax_ (R^2^ = 0.51; Fig 3E), whereas PLSR model predictive ability was substantially reduced for J_max_ (R^2^ = 0.24; Fig 3F). The RSME was the same for both models (RMSE = 7.7; Fig 3E and 3F).

To compare the relative significance of different wavelengths in a given PLSR model, the selectivity ratio (SR) was used as a method of variable selection. The SR was used to assess relative contributions of different portions of the spectrum to the overall PLSR model. Both V_cmax_ and J_max_ models were sensitive to variation in the visible wavelength and near infrared (Fig 2B, Fig 2C). Peak SR was at 703 nm for V_cmax_ and 583 nm for J_max_ (Fig 2B and 2C). Contributions from the short-wave infrared regions were small to both V_cmax_ and J_max_ models, however the SWIR contributed more to the J_max_ models than V_cmax_ models (Fig 2B and 2C).

**Fig 4:**
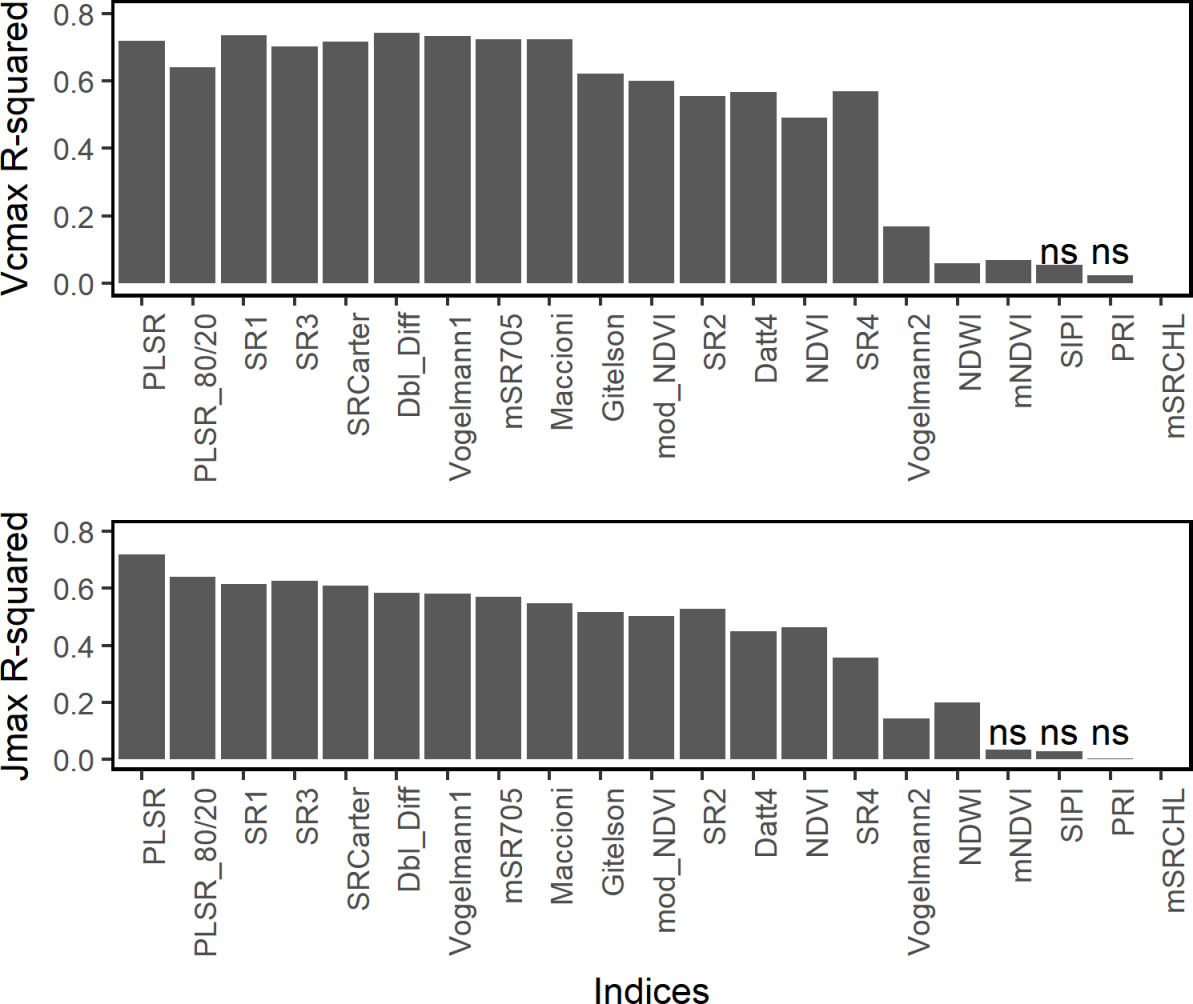
Comparison of predictive abilities of hyperspectral indices and PLSR models. Predictive capability for (A) maximum rates of RuBP carboxylation (V_cmax_, μmol m^-2^ s-^1^) and (B) RuBP regeneration J_max_ (μmol m^-2^ s-^1^). Pairwise correlations between V_cmax_ and J_max_ estimated using standard gas exchange techniques and spectral indices were tested and R^2^ is reported. The R^2^ reported for the PLSR model is from the leave-one-out cross-validation approach. Indices with non-significant correlations are represented with “ns”.

To assess relationships between existing hyperspectral indices and photosynthetic capacity, correlations between V_cmax_ and J_max_ and a suite of hyperspectral vegetation indices were compared (Fig 4). Estimated V_cmax_ and J_max_ from hyperspectral chlorophyll and stress indices were well-correlated with measured values (Table 1). For V_cmax_, four other metrics were all comparable to the full PLSR model (R^2^ = 0.72) based on the coefficient of determination: Maccioni (R^2^ = 0.72), Double Difference (R^2^ = 0.74), Vogelmann2 (R^2^ = 0.73), and SR1 (R^2^ = 0.74) (Fig 4A). The PLSR model for J_max_ (R^2^ = 0.72) outperformed all tested hyperspectral indices, the best of which was SR3 (R^2^ = 0.63; Fig 4B). Several indices had very low or non-significant correlations with V_cmax_, including PRI, NDWI, mNDVI, and SIPI (Table 1). The mNDVI, SIPI, PRI, and mSRCHL indices all had non-significant relationships with Jmax (Table 1). The hyperspectral normalized difference vegetation index (NDVI) had moderate predictive capability for Vcmax (R^2^ = 0.49) and J_max_ (R^2^ = 0.46). The MODIS-like NDVI (“mod_NDVI”) performed better than the hyperspectral NDVI for both V_cmax_ (R^2^ = 0.60) and J_max_ (R^2^ = 0.50) (Fig 4, Table 1).

## Discussion

Our results show that hypers can predict photosynthetic parameters across time, suggesting that these hyperspectral remote sensing techniques have great potential to constrain model estimates of plant function. In this study, relationships between leaf reflectance spectra and photosynthetic capacity were robust throughout a 7-week period with dynamic change in photosynthetic capacity. Hyperspectral vegetation indices to estimate chlorophyll content were correlated with the key determinants of photosynthetic capacity, V_cmax_ and J_max_. Predictions of V_cmax_ and J_max_ from PLSR models derived from leaf reflectance spectra were proportionally-sensitive to observed variation in V_cmax_ and J_max_. These results support our hypothesis that PLSR models that utilize the full spectrum can predict photosynthetic capacity through time. These findings highlight the potential of hyperspectral remote sensing methods to accurately predict V_cmax_ and J_max_ despite dynamic temporal variation in photosynthetic capacity related to within-season variation and plant stress.

Leaf-level hyperspectral data have previously been used to estimate photosynthesis [66] and photosynthetic capacity across temperature regimes in glasshouse experiments [32], and in diverse agroecosystems [41,42], but our study is the first to assess the impacts of *in situ* within-season temporal variation on estimates of photosynthetic capacity derived from leaf reflectance spectra. As in previous studies, measured and PLSR-modeled leaf traits were significantly correlated [32,42,66,67]. Regression models predicted V_cmax_ and J_max_ during a 7-week period with dynamic declines in photosynthetic capacity. This is confirmation that the spectral signals detected by leaf reflectance observations are accurately tracking seasonal metabolic adjustments made within the photosynthetic machinery of the leaf. When PLSR models were trained on only 80% of the data and tested on the remaining 20%, their mean predictive capability were similar to that of the full PLSR models. This shows that relationships between leaf reflectance spectra and V_cmax_ and J_max_ are robust despite large variation in these values. Known hyperspectral vegetation indices had varying predictive capabilities. Hyperspectral vegetation indices developed for estimating chlorophyll content (e.g. SR1, Double Difference, Vogelmann 1) and plant stress (SRCarter) had the highest correlations with plant photosynthetic capacity (Table 1). The wavelengths that explained variance in photosynthetic capacity in the PLSR models (based on selectivity ratio) were consistent with the wavelengths that comprised the best-performing hyperspectral indices. The important wavelengths in PLSR models, based on Selectivity Ratio, for predicting V_cmax_ and J_max_ largely fell in the visible and short wavelength end of the near-infrared (~500 to 850 nm), with minor contributions from wavelengths in the short-wave infrared. Important wavelengths in the visible region fell largely in the blue region (450–495 nm) and red regions (620–650 nm) for the V_cmax_ models, which is consistent with the chlorophyll absorption regions (i.e. ∼430–460 nm and 640–670 nm). The highest-performing hyperspectral vegetation indices generally leveraged diagnostic differences in the red-edge portion (680–750 nm) of the spectrum to estimate chlorophyll content. The red edge region of reflectance is known to be sensitive to differences in chlorophyll content, and chlorophyll content is generally positively correlated with photosynthetic capacity [68]. Overall, both hyperspectral indices and PLSR models had the capability to predict variation in within-season photosynthetic capacity.

Accurate representation of photosynthesis in terrestrial biosphere models is essential to predicting future carbon and global change dynamics [69]. Although modeled rates of photosynthesis are sensitive to V_cmax_ and J_max_ [3,70,71], most terrestrial biosphere models use static values for these parameters [27]. Furthermore, V_cmax_ and J_max_ values are often parameterized based on limited or poorly represented data sets [27]. Monthly optimization of V_cmax_ improved process-based biosphere model (Organizing Carbon and Hydrology in Dynamic Ecosystems; ORCHIDEE) representation of seasonal carbon dynamics (NEE; Net Ecosystem Exchange) in a tropical evergreen forest in Brazil, however, seasonal parameter variations could not be extrapolated spatially [28]. Remote sensing observations can improve model representation of photosynthesis across spatial and temporal scales [31,55,72]. Our results support the use of remotely sensed estimation of photosynthetic capacity using hyperspectral observations. Furthermore, hyperspectral remote sensing could be used to incorporate spatially explicit photosynthetic capacity/environment relationships in next-generation trait-based models. Our results highlight the potential of hyperspectral remote sensing to parameterize determinants of photosynthetic capacity and inform trait-environment relationships in terrestrial biosphere models, thereby improving model representation of photosynthesis and carbon dynamics.

Although PLSR model predictive capabilities for V_cmax_ and J_max_ were generally similar, there were differences in PLSR model performance and sensitivity between the two variables. The mean predictive capability of the 80% PLSR models were comparable for V_cmax_ and J_max_ (Table 1); however, the variance in J_max_ was nearly twice that of the variance in V_cmax_ (Table 1, S1 Fig). A rigorous test of PLSR model performance in the population with individual variation in drought stress indicated modest reductions in predictive capability for V_cmax_ and substantial reductions in predictive capability for J_max_. This test of temporal stability indicates that predictive relationships between leaf reflectance spectra and photosynthetic capacity are reduced when there is within-population variation in environmental stress, particularly for J_max_. PLSR models for J_max_ included more wavelengths in the short-wave infrared that are associated with leaf water content and internal structure. The performance improvement of the PLSR model for J_max_ compared to simple indices underscores the importance of full spectral information for predicting this parameter (Fig 4B)[42]. Terrestrial biosphere models generally simulate J_max_ as a function of V_cmax_ rather than as its own parameter [73]. However, our results suggest that V_cmax_ and J_max_ may be differentially sensitive to within-population variation in environmental stress.

Quantifying temporal variation in relationships between photosynthetic capacity and leaf reflectance spectra is timely given the increasing availability of high resolution spectral remote sensing, such as hyperspectral overflights planned by the National Ecological Observatory Network (NEON) [74] and NASA’s Hyperspectral Infrared Imager (HyspIRI) mission [75]. NEON hyperspectral overflights, which may only occur once per year over a given region, may be used to develop predictive relationships between leaf reflectance spectra and photosynthetic capacity. Our results suggest that such predictive relationships developed from data in a short portion of the growing season could hold true throughout the growing season. The widely used NDVI had moderate predictive capacity for V_cmax_ and J_max_. Notably, our approximation of MODIS NDVI had higher predictive capacity than hyperspectral NDVI (Table 1). Detecting temporal changes in photosynthetic capacity using MODIS NDVI could improve model predictions of photosynthesis given its broad spatial and daily temporal coverage. Another important consideration in extrapolating the results of this study to aerial and satellite remote sensing is additional technical challenges posed by these approaches, such as view angle effects, canopy architecture, and atmospheric effects. More studies are needed to further test temporal stability of relationships between leaf reflectance spectra and photosynthetic capacity in other contexts before relationships from a single hyperspectral overflight can be extrapolated through the growing season. Nonetheless, our results suggest promise for this approach.

In this study, spectral estimation of V_cmax_ and J_max_ in hybrid poplar was robust to temporal variation of up to 200% in photosynthetic capacity. Applying remote sensing tools to predict photosynthetic capacity across a wider range of wildlands requires an *in situ* test of this method outside of agroecosystem or controlled glasshouse conditions across a time period of variable abiotic and biotic conditions. Our results show that relationships between photosynthetic capacity and leaf reflectance spectra developed from limited data can in some cases be extrapolated temporally. These results highlight the potential of hyperspectral remote sensing methods to detect dynamic temporal variations in V_cmax_ and J_max_ related to seasonality and plant stress, thereby aiding improved estimates of plant productivity and associated carbon budget. Furthermore, our results suggest that terrestrial biosphere models could use hyperspectral remote sensing to parameterize V_cmax_ and J_max_ within season to improve predictions of future carbon dynamics. Reliable and precise methods to estimate V_cmax_ and J_max_ across spatial and temporal scales will improve understanding of ecosystem carbon uptake and the terrestrial carbon sink.

## Supporting information

S1 Fig Variance of R^2^ values based on proportion of data used for training the PLSR model. Each point represents the r-squared between predicted and actual Vcmax/Jmax values from PLSR using a random sample corresponding to the designated proportion of training data (each proportion was sampled 100 times).
(TIF)

S1 Table Mean, median, and standard deviation of R^2^ values based on proportion of data used for training the PLSR model. The mean, median, and standard deviation in R^2^ of 100 PLSR models per training proportion are represented.
(DOCX)
