# Supplementary figures and images for: Beyond greenness: Detecting temporal changes in photosynthetic capacity with hyperspectral reflectance data
Source: PLoS One. 2017 Dec 27;12(12):e0189539. doi: 10.1371/journal.pone.0189539 (PMC5744967; doi:10.1371/journal.pone.0189539)

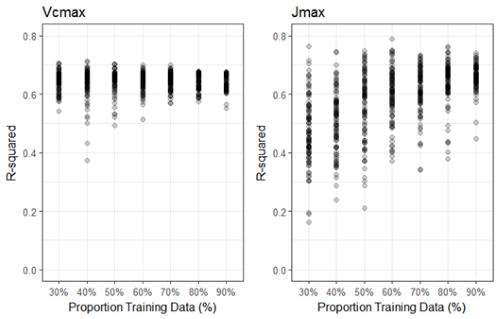

Supplement: S1 Fig — Each point represents the r-squared between predicted and actual Vcmax/Jmax values from PLSR using a random sample corresponding to the designated proportion of training data (each proportion was sampled 100 times). (TIF) [file pone.0189539.s001.tif]
