# Supplementary material for: Beyond greenness: Detecting temporal changes in photosynthetic capacity with hyperspectral reflectance data
Source: PLoS One. 2017 Dec 27;12(12):e0189539. doi: 10.1371/journal.pone.0189539 (PMC5744967; doi:10.1371/journal.pone.0189539)
Supplement: S1 Table — The mean, median, and standard deviation in R2 of 100 PLSR models per training proportion are represented. (DOCX) [file pone.0189539.s002.docx]

S1 Table. Mean, median, and standard deviation of R^2^ values based on proportion of data used for training the PLSR model.

|  | **Vcmax** |  |  |
| --- | --- | --- | --- |
| Prop. Training | Mean R^2^ | Median R^2^ | Standard Deviation |
| 30% | 0.65 | 0.65 | 0.040 |
| 40% | 0.64 | 0.65 | 0.042 |
| 50% | 0.64 | 0.65 | 0.037 |
| 60% | 0.64 | 0.65 | 0.033 |
| 70% | 0.64 | 0.65 | 0.031 |
| 80% | 0.64 | 0.65 | 0.032 |
| 90% | 0.64 | 0.64 | 0.030 |
|  | **Jmax** |  |  |
| Prop. Training | Mean R^2^ | Median R^2^ | Standard Deviation |
| 30% | 0.48 | 0.48 | 0.11 |
| 40% | 0.53 | 0.53 | 0.11 |
| 50% | 0.57 | 0.59 | 0.11 |
| 60% | 0.60 | 0.62 | 0.11 |
| 70% | 0.62 | 0.65 | 0.10 |
| 80% | 0.64 | 0.66 | 0.066 |
| 90% | 0.65 | 0.65 | 0.042 |

The mean, median, and standard deviation in R^2^ of 100 PLSR models per training proportion are represented.
